# Supplementary material for: Risk scores for predicting HIV incidence among adult heterosexual populations in sub‐Saharan Africa: a systematic review and meta‐analysis
Source: J Int AIDS Soc. 2022 Jan 9;25(1):e25861. doi: 10.1002/jia2.25861 (PMC8743366; doi:10.1002/jia2.25861)
Supplement: Supplementary file 1 — Appendix I. Database search strategy Appendix II. Details on data extraction Table S1. Characteristics of the included studies Table S2. Methods for assessing curable sexually transmitted infections (STIs) and HSV‐2 status Table S3. Summary table for the risk of bias assessment according to the PROBAST checklist Table S4. Summary table on the concerns for applicability according to the PROBAST checklist Table S5. Summary of missing data and loss‐to‐follow‐up Table S6. Summary adjusted and unadjusted hazard ratios (HRs) Table S7. HIV incidence and distribution of high‐risk group by each risk score Figure S1. Risk of bias assessment (a) and concerns for applicability (b) for the model development (i) and validation (ii) studies. Appendix III. PRIMSA 2020 Abstract Checklist Appendix IV. PRISMA 2020 Main Checklist [file JIA2-25-e25861-s001.pdf]

Risk scores for predicting HIV incidence among adult heterosexual populations in sub-Saharan Africa: a systematic review and meta-analysis—Supplementary Materials

Katherine M Jia, Hallie Eilerts, Olanrewaju Edun, Kevin Lam, Adam T Howes, Matthew L Thomas, Jeffrey W Eaton

Contents

Appendix I Database search strategy 2

Appendix II. Details on data extraction 5

Table S1. Characteristics of the included studies 6

Table S2. Methods for assessing curable sexually-transmitted infections (STIs) and HSV-2 status. 13

Table S3. Summary table for the risk of bias assessment according to the PROBAST checklist 15

Table S4. Summary table on the concerns for applicability according to the PROBAST checklist 16

Table S5. Summary of missing data and loss-to-follow-up 17

Table S6. Summary adjusted and unadjusted hazard ratios (HRs) 18

Table S7. HIV incidence and distribution of high-risk group by each risk score 19

Figure S1. Risk of bias assessment (A) and concerns for applicability (B) for the model development (i) and validation (ii) studies. 24

Appendix III. PRIMSA 2020 Abstract Checklist 25

Appendix IV. PRISMA 2020 Main Checklist 26

References 29

Appendix I Database search strategy
Search date: 15th February 2021 (Monday)

1. MEDLINE via Ovid

	Query
**1**	((risk* adj appraisal*) or (risk* adj algorithm*) or (risk* adj “assessment tool”) or (risk* adj1 calculat*) or (risk* adj chart*) or (risk* adj1 checklist*) or (risk* adj “classification tool”) or (risk* adj disk) or (risk* adj disc?) or (risk* adj function*) or (risk* adj equation*) or (risk* adj1 index) or (risk* adj1 indices) or (risk* adj3 scale*) or (risk* adj3 scor*) or (risk* adj “stratification tool”) or (risk* adj table*) or (risk* adj threshold*) or (risk* adj3 tool*) or (risk* adj prediction*) or ("risk assessment" adj function*) or (prognostic adj tool) or (prognostic adj model) or ((risk or inciden* or hazard* or prognos*) and ((scor* adj algorithm*) or (scor* adj scheme*) or (scor* adj system*) or (scor* adj tool*) or (screening adj score*) or (prediction adj equation) or (predicti* adj instrument*) or (predicti* adj model*) or (predicti* adj rule) or (predicti* adj scor*) or (projecti* adj1 risk*)))).tw
**2**	exp decision support techniques/ or exp clinical decision rules/ or exp data interpretation, statistical/ [included all subheadings]
**3**	exp Nomograms/
**4**	OR # 2-#3
**5**	exp algorithms/ or exp artificial intelligence/ or exp latent class analysis/
**6**	(screen* or scor* or predict* or risk* or prognos*).tw.
**7**	AND #5 - #6
**8**	OR #1, #4, #7
**9**	(Africa* or Angola or Benin or Botswana or “Burkina Faso” or Burundi or “Cabo Verde” or “Cape Verde” or Cameroon or “Central African Republic” or Chad or Comoros or “Democratic Republic of Congo” or DRC or “Republic of Congo” or “Cote d'Ivoire” or “Cote D' Ivoire” or “Ivory Coast” or Djibouti or “Equatorial Guinea” or Eritrea or Eswatini or Ethiopia or Gabon or Gambia or Ghana or Guinea or Guinea-Bissau or Kenya or Lesotho or Liberia or Madagascar or Malawi or Mali or Mauritania or Mauritius or Mozambique or Namibia or Niger or Nigeria or Rwanda or (“Sao Tome” adj1 Princip*) or Senegal or Seychelles or “Sierra Leone” or Somalia or “South Africa” or “South Sudan” or Sudan or Swaziland or Tanzania or Togo or Uganda or Zambia or Zimbabwe).mp
**10**	(HIV* or “human immunodeficiency virus” or “human-immunodeficiency-virus” or AID* or “Acquired Immunodeficiency Syndrome”).ti.
**11**	AND # 8 - #10

2. Embase, MIDIRS, APA PsycInfo, Global Health via Ovid

	Query
**1**	((risk* adj appraisal*) or (risk* adj algorithm*) or (risk* adj “assessment tool”) or (risk* adj1 calculat*) or (risk* adj chart*) or (risk* adj1 checklist*) or (risk* adj “classification tool”) or (risk* adj disk) or (risk* adj disc?) or (risk* adj function*) or (risk* adj equation*) or (risk* adj1 index) or (risk* adj1 indices) or (risk* adj3 scale*) or (risk* adj3 scor*) or (risk* adj “stratification tool”) or (risk* adj table*) or (risk* adj threshold*) or (risk* adj3 tool*) or (risk* adj prediction*) or ("risk assessment" adj function*) or (prognostic adj tool) or (prognostic adj model) or ((risk or inciden* or hazard* or prognos*) and ((scor* adj algorithm*) or (scor* adj scheme*) or (scor* adj system*) or (scor* adj tool*) or (screening adj score*) or (prediction adj equation) or (predicti* adj instrument*) or (predicti* adj model*) or (predicti* adj rule) or (predicti* adj scor*) or (projecti* adj1 risk*)))).tw
**2**	(Africa* or Angola or Benin or Botswana or “Burkina Faso” or Burundi or “Cabo Verde” or “Cape Verde” or Cameroon or “Central African Republic” or Chad or Comoros or “Democratic Republic of Congo” or DRC or “Republic of Congo” or “Cote d'Ivoire” or “Cote D' Ivoire” or “Ivory Coast” or Djibouti or “Equatorial Guinea” or Eritrea or Eswatini or Ethiopia or Gabon or Gambia or Ghana or Guinea or Guinea-Bissau or Kenya or Lesotho or Liberia or Madagascar or Malawi or Mali or Mauritania or Mauritius or Mozambique or Namibia or Niger or Nigeria or Rwanda or (“Sao Tome” adj1 Princip*) or Senegal or Seychelles or “Sierra Leone” or Somalia or “South Africa” or “South Sudan” or Sudan or Swaziland or Tanzania or Togo or Uganda or Zambia or Zimbabwe).mp
**3**	(HIV* or “human immunodeficiency virus” or “human-immunodeficiency-virus” or AID* or “Acquired Immunodeficiency Syndrome”).ti.
**4**	AND # 1 - #3
**5**	remove duplicates from # 4

3. Scopus

	Query
**1**	TITLE-ABS-KEY ((risk* W/0 appraisal*) or (risk* W/0 algorithm*) or (risk* W/0 “assessment tool”) or (risk* W/1 calculat*) or (risk* W/0 chart*) or (risk* W/1 checklist*) or (risk* W/0 “classification tool”) or (risk* W/0 disk) or (risk* W/0 disc?) or (risk* W/0 function*) or (risk* W/0 equation*) or (risk* W/1 index) or (risk* W/1 indices) or (risk* W/3 scale*) or (risk* W/3 scor*) or (risk* W/0 “stratification tool”) or (risk* W/0 table*) or (risk* W/0 threshold*) or (risk* W/3 tool*) or (risk* W/0 prediction*) or ("risk assessment" W/0 function*) or (prognostic W/0 tool) or (prognostic W/0 model) or ((risk OR inciden* OR hazard* OR prognos*) and ((scor* W/0 algorithm*) or (scor* W/0 scheme*) or (scor* W/0 system*) or (scor* W/0 tool*) or (screening W/0 score*) or (prediction W/0 equation) or (predicti* W/0 instrument*) or (predicti* W/0 model*) or (predicti* W/0 rule) or (predicti* W/0 scor*) or (projecti* W/0 risk*))))
**2**	ALL (Africa* or Angola or Benin or Botswana or “Burkina Faso” or Burundi or “Cabo Verde” or “Cape Verde” or Cameroon or “Central African Republic” or Chad or Comoros or “Democratic Republic of Congo” or DRC or “Republic of Congo” or “Cote d'Ivoire” or “Cote D' Ivoire” or “Ivory Coast” or Djibouti or “Equatorial Guinea” or Eritrea or Eswatini or Ethiopia or Gabon or Gambia or Ghana or Guinea or Guinea-Bissau or Kenya or Lesotho or Liberia or Madagascar or Malawi or Mali or Mauritania or Mauritius or Mozambique or Namibia or Niger or Nigeria or Rwanda or (“Sao Tome” W/1 Princip*) or Senegal or Seychelles or “Sierra Leone” or Somalia or “South Africa” or “South Sudan” or Sudan or Swaziland or Tanzania or Togo or Uganda or Zambia or Zimbabwe)
**3**	TITLE(HIV* or "human immunodeficiency virus" or "human-immunodeficiency-virus" or AID* or "Acquired Immunodeficiency Syndrome")
**4**	AND # 1 - #3

4. CINAHL (EBSCO)

	Query
**1**	Title, Abstract, Subject Headings and Keywords:<br>((risk* N0 appraisal*) or (risk* N0 algorithm*) or (risk* N0 “assessment tool”) or (risk* N1 calculat*) or (risk* N0 chart*) or (risk* N1 checklist*) or (risk* N0 “classification tool”) or (risk* N0 disk) or (risk* N0 disc?) or (risk* N0 function*) or (risk* N0 equation*) or (risk* N1 index) or (risk* N1 indices) or (risk* N3 scale*) or (risk* N3 scor*) or (risk* N0 “stratification tool”) or (risk* N0 table*) or (risk* N0 threshold*) or (risk* N3 tool*) or (risk* N0 prediction*) or ("risk assessment" N0 function*) or (prognostic N0 tool) or (prognostic N0 model) or ((risk OR inciden* OR hazard* OR prognos*) AND ((scor* N0 algorithm*) or (scor* N0 scheme*) or (scor* N0 system*) or (scor* N0 tool*) or (screening N0 score*) or (prediction N0 equation) or (predicti* N0 instrument*) or (predicti* N0 model*) or (predicti* N0 rule) or (predicti* N0 scor*) or (projecti* N0 risk*))))
**2**	(MH "Clinical Prediction Rules")
**3**	1 OR 2
**4**	All texts:<br>(Africa* or Angola or Benin or Botswana or “Burkina Faso” or Burundi or “Cabo Verde” or “Cape Verde” or Cameroon or “Central African Republic” or Chad or Comoros or “Democratic Republic of Congo” or DRC or “Republic of Congo” or “Cote d'Ivoire” or “Cote D' Ivoire” or “Ivory Coast” or Djibouti or “Equatorial Guinea” or Eritrea or Eswatini or Ethiopia or Gabon or Gambia or Ghana or Guinea or Guinea-Bissau or Kenya or Lesotho or Liberia or Madagascar or Malawi or Mali or Mauritania or Mauritius or Mozambique or Namibia or Niger or Nigeria or Rwanda or (“Sao Tome” N1 Princip*) or Senegal or Seychelles or “Sierra Leone” or Somalia or “South Africa” or “South Sudan” or Sudan or Swaziland or Tanzania or Togo or Uganda or Zambia or Zimbabwe)
**6**	Title<br>HIV* or "human immunodeficiency virus" or "human-immunodeficiency-virus" or AID* or "Acquired Immunodeficiency Syndrome"
**7**	AND # 3 - # 6

5. Cochrane

	Query
**1**	Title / Abstract / Keyword: ((risk* NEXT appraisal*) or (risk* NEXT algorithm*) or (risk* NEXT “assessment tool”) or (risk* NEAR/1 calculat*) or (risk* NEXT chart*) or (risk* NEAR/1 checklist*) or (risk* NEXT “classification tool”) or (risk* NEXT disk) or (risk* NEXT disc?) or (risk* NEXT function*) or (risk* NEXT equation*) or (risk* NEAR/1 index) or (risk* NEAR/1 indices) or (risk* NEAR/3 scale*) or (risk* NEAR/3 scor*) or (risk* NEXT “stratification tool”) or (risk* NEXT table*) or (risk* NEXT threshold*) or (risk* NEAR/3 tool*) or (risk* NEXT prediction*) or ("risk assessment" NEXT function*) or (prognostic NEXT tool) or (prognostic NEXT model) or ((risk OR inciden* OR hazard* OR prognos*) and ((scor* NEXT algorithm*) or (scor* NEXT scheme*) or (scor* NEXT system*) or (scor* NEXT tool*) or (screening NEXT score*) or (prediction NEXT equation) or (predicti* NEXT instrument*) or (predicti* NEXT model*) or (predicti* NEXT rule) or (predicti* NEXT scor*) or (projecti* NEXT risk*))))
**2**	All text: (Africa* or Angola or Benin or Botswana or “Burkina Faso” or Burundi or “Cabo Verde” or “Cape Verde” or Cameroon or “Central African Republic” or Chad or Comoros or “Democratic Republic of Congo” or DRC or “Republic of Congo” or “Cote d'Ivoire” or “Cote D' Ivoire” or “Ivory Coast” or Djibouti or “Equatorial Guinea” or Eritrea or Eswatini or Ethiopia or Gabon or Gambia or Ghana or Guinea or Guinea-Bissau or Kenya or Lesotho or Liberia or Madagascar or Malawi or Mali or Mauritania or Mauritius or Mozambique or Namibia or Niger or Nigeria or Rwanda or (“Sao Tome” NEAR/1 Princip*) or Senegal or Seychelles or “Sierra Leone” or Somalia or “South Africa” or “South Sudan” or Sudan or Swaziland or Tanzania or Togo or Uganda or Zambia or Zimbabwe)
**3**	Record title: (HIV* or "human immunodeficiency virus" or "human-immunodeficiency-virus" or AID* or "Acquired Immunodeficiency Syndrome")
**4**	1 AND 2 AND 3

6. Web of Science

	Query
**1**	TS = ((risk* NEAR/0 appraisal*) or (risk* NEAR/0 algorithm*) or (risk* NEAR/0 “assessment tool”) or (risk* NEAR/1 calculat*) or (risk* NEAR/0 chart*) or (risk* NEAR/1 checklist*) or (risk* NEAR/0 “classification tool”) or (risk* NEAR/0 disk) or (risk* NEAR/0 disc?) or (risk* NEAR/0 function*) or (risk* NEAR/0 equation*) or (risk* NEAR/1 index) or (risk* NEAR/1 indices) or (risk* NEAR/3 scale*) or (risk* NEAR/3 scor*) or (risk* NEAR/0 “stratification tool”) or (risk* NEAR/0 table*) or (risk* NEAR/0 threshold*) or (risk* NEAR/3 tool*) or (risk* NEAR/0 prediction*) or ("risk assessment" NEAR/0 function*) or (prognostic NEAR/0 tool) or (prognostic NEAR/0 model) or ((risk OR inciden* OR hazard* OR prognos*) and ((scor* NEAR/0 algorithm*) or (scor* NEAR/0 scheme*) or (scor* NEAR/0 system*) or (scor* NEAR/0 tool*) or (screening NEAR/0 score*) or (prediction NEAR/0 equation) or (predicti* NEAR/0 instrument*) or (predicti* NEAR/0 model*) or (predicti* NEAR/0 rule) or (predicti* NEAR/0 scor*) or (projecti* NEAR/0 risk*))))
**2**	ALL = (Africa* or Angola or Benin or Botswana or “Burkina Faso” or Burundi or “Cabo Verde” or “Cape Verde” or Cameroon or “Central African Republic” or Chad or Comoros or “Democratic Republic of Congo” or DRC or “Republic of Congo” or “Cote d'Ivoire” or “Cote D' Ivoire” or “Ivory Coast” or Djibouti or “Equatorial Guinea” or Eritrea or Eswatini or Ethiopia or Gabon or Gambia or Ghana or Guinea or Guinea-Bissau or Kenya or Lesotho or Liberia)
**3**	ALL = (Madagascar or Malawi or Mali or Mauritania or Mauritius or Mozambique or Namibia or Niger or Nigeria or Rwanda or (“Sao Tome” AND Princip*) or Senegal or Seychelles or “Sierra Leone” or Somalia or “South Africa” or “South Sudan” or Sudan or Swaziland or Tanzania or Togo or Uganda or Zambia or Zimbabwe)
**4**	OR #2 - #3
**5**	TI = (HIV* or "human immunodeficiency virus" or "human-immunodeficiency-virus" or AID* or "Acquired Immunodeficiency Syndrome")
**6**	AND # 1, #4, #5

Appendix II. Details on data extraction

The following items are extracted from the included studies:

**Study**	1. Study design<br>2. Study period<br>3. Countries of study
**Participants**	1. Number and location of sites,<br>2. Inclusion/exclusion criteria<br>3. Participant description<br>4. Intervention(s) received (if any) and effectiveness
**Sample size**	1. Number of participants enrolled<br>2. Number of participants analysed<br>3. Reason(s) for exclusion of enrolled participants from analysis<br>4. Number of HIV incident cases<br>5. Number of HIV incident cases per candidate predictor (Events Per Variable)
**Missing data**	1. Number of participants with any missing values (predictors)<br>2. Loss-to-follow-up (i.e., absence of at least one follow-up HIV test)<br>3. Method for handling missing data
**Outcomes**	1. Definition and method for measurement of outcome<br>2. Was the same outcome definition (and method for measurement) used in all patients?<br>3. Type of outcome (e.g., single or combined endpoints)<br>4. Was the outcome assessed without knowledge of the candidate predictors (i.e., blinded)?<br>5. Were candidate predictors part of the outcome (e.g., in panel or consensus diagnosis)?<br>6. Time point where the outcome is determined<br>7. Total duration of follow-up<br>8. HIV incidence
**Predictors**	1. Timing of predictor measurement<br>2. Number and type of predictors<br>3. Methods for measurement of sexually transmitted infections (STIs)<br>4. Were predictors assessed blinded for outcome, and for each other (if relevant)?<br>5. Handling of predictors in the modelling (e.g., continuous, linear,<br>6. non-linear transformations or categorised)
**Model development**	1. Modelling approach<br>2. Modelling assumptions satisfied<br>3. Method for selection of predictors for inclusion in multivariable modelling<br>4. Method for selection of predictors during multivariable modelling<br>5. Shrinkage of predictor weights or regression coefficients
**Model performance assessment**	1. Calibration and discrimination measures that have been used<br>2. Classification measures and how cut points have been determined
**Model evaluation**	1. Method used for testing model performance: internal validation (e.g., bootstrapping, cross-validation or none) or external validation<br>2. In case of poor validation, whether model was adjusted or updated
**Results**	1. Predictors retained in the final and other multivariable models (e.g., excluding the laboratory diagnosed STIs)<br>2. Coefficients for the predictors (i.e., effect size estimates)<br>3. Model performance measures (with confidence intervals)<br>4. Comparison of the distribution of predictors (including missing data) for development and validation datasets

Table S1. Characteristics of the included studies

First author (Year);	Any intervention; if yes, is the intervention effective and adjusted in the model?	Description of dataset; Inclusion / exclusion criteria	Age	Timing of outcome determination	Candidate predictors	Predictors selected for inclusion into the model	Predictors retained in the final model
**(I) Women only (All ages / 25+years old)**							
**Wand (2012) [1]**	**MIRA** [2]<br>**Intervention**:<br>Latex diaphragm, lubricant gel, and condoms (intervention) vs condoms alone (control)<br><br>**Outcome:**<br>Prevention of heterosexual HIV acquisition among women<br><br>**Any significant effect(s):**<br>No. The risk score was developed by using the Durban data only. The intervention did not show a significant effect in reducing HIV incidence in the Durban sites (Hazard Ratio [HR]: 0.95 [0.69, 1.31]).<br><br>**Intervention incorporated as a predictor in the model?**<br>No. Only contraception uses at baseline and condom use in the past 3 months before enrolment were included.	Randomised controlled trial (RCT);<br><br>(i) sexually active women,<br><br>(ii) willing to use contraception / not planning to get pregnant in the next 24 months from two sites,<br><br>(iii) residing in Durban, South Africa	**Mean**: 27;<br><br>**IQR**: 22-34	**Outcome determination**: Quarterly followed-up with a total period ranging from 12 to 24 months<br><br>**Prediction horizon:**<br>any event during the entire follow-up period	• Age<br>• Cohabitation status (whether the participant was living with her sexual partner)<br>• Level of education<br>• Employment status<br>• Number of lifetime sexual partners<br>• Age at first sex<br>• Consistent condom use (in past three months)<br>• Contraception use [long term (tubal ligation, vasectomy, intrauterine device), hormonal injectables, oral contraceptives), barrier methods (male/female condoms)]<br>• Average number of weekly sex acts<br>• Partner risk (defined as one or more of: any sexual partners testing positive for HIV;<br>• suspecting or knowing that their regular partner had other sex partners in the past three months; any vaginal sex when partner was under influence of drugs or alcohol in past three months; regular partner was away from home for one or more months)<br>• High behavioural risk (defined as one or more of: any exchange of sex for money, food, drugs, or shelter; two or more sexual partners within past three months; ever having vaginal sex under influence of drugs or alcohol in past three months; ever using a needle for injectable drug use; having anal sex in past three months)<br>• Any STIs (Neisseria gonorrhoeae, Chlamydia<br>• trachomatis and syphilis)<br>• Herpes simplex virus<br>• 2 (HSV-2) in the past six months<br>• Any reproductive tract infections (candidiasis, bacterial vaginosis),<br>• Genital epithelial disruption<br>• Genital signs<br>• Genital discharge<br>• Genital ulcer<br>• Abnormal vulva	**Criteria for variable to be included**: univariate association (P threshold not mentioned)<br><br>• Age (<25, 25-34, 35+ yrs)<br>• Lifetime male sexual partners<br>• Behaviour risk<br>• Coital frequency (per week)<br><br>• Cohabiting with a sex partner<br>• Genital epithelial disruption<br>• Genital signs<br>• Genital discharge<br>• Genital Ulcer<br>• Abnormal vulva	**Cox regression**;<br>Stepwise backward elimination procedures<br><br>**Final model:**<br>• Lifetime male sexual partners<br>• Behavioural risk<br>• Cohabiting with a sexual partner<br>• Genital epithelial disruption<br>• Genital discharge
**Wand (2018) [3]**	The risk score was developed based on KwaZulu Natal, South Africa only. At all visits, the participants received counselling on risk reduction and had access to male condoms as desired [3].<br><br>**MIRA**[2]<br>**Intervention**:<br>Latex diaphragm, lubricant gel, and condoms (intervention) vs condoms alone (control)<br><br>**Outcome:**<br>Prevention of heterosexual HIV acquisition among women<br><br>**Any significant effect(s):**<br>No. The intervention did not show a significant effect in reducing HIV incidence in the KZN (Durban) sites (adjusted HR: 0.95 [0.69, 1.31]).	RCTs;<br><br>(i) sexually active women,<br><br>(ii) willing to use contraception / not planning to get pregnant,<br><br>(iii) residing in KwaZulu Natal, South Africa.	**Median** 27; **IQR:** 22–33	**Outcome determination**: Various timepoint depending on studies<br><br>**Prediction horizon:**<br>any event during the entire follow-up period	• Age (<20, 20–24, 25–29, 30–34, 35–39 and 40+ years);<br>• Married/cohabiting with a sexual partner (yes/no)<br>• Level of education (less than high school vs. completed high school or above)<br>• Number of sexual partners in past three months (3+ vs.<3);<br>• age at sexual debut (<16 vs. 16+ years),<br>• condom used at last sex (yes/no).<br>• Injectables (yes/no),<br>• oral contraceptives (pills) (yes/no)<br>• male condom (yes/no);<br>• parity (null/primiparity: <2 births, multi-parity: 2 vs. multi-parity: 3+)<br>• diagnosed with STIs (chlamydia, gonorrhoea, syphilis)<br>• language spoken at home,<br>• employment/regular income (yes vs. no),<br>• partner's circumcision status<br>• average number of sexual acts in the past 7 days.)	**Predictors with univariate association P<0.05 were included.**<br><br>**Variables included:**<br>• Age<br>• Age at sexual debut<br>• education,<br>• employment,<br>• partner's circumcision status,<br>• condom not used in last sex<br>• Married/cohabiting with a sexual partner<br>• Number of sexual partners<br>• Parity<br>• Injectable contraception<br>• Diagnosed with STIs	**Cox regression**.<br>Stepwise backward elimination procedures<br><br>**Final model:**<br>• Age<br>• Age at sexual debut<br>• Married/cohabiting with a sexual partner<br>• Number of sexual partners<br>• Parity<br>• Injectable contraception<br>• Diagnosed with STIs

**MDP 301 [4]**<br>**Intervention:**<br>2% PRO2000, 0.5% PRO2000, or placebo gel groups.<br><br>At the 12, 24, 40 and 52 week clinic visits, women were provided with HIV testing and counselling with promotion of safer sex practices, provision of free condoms, and diagnosis and treatment of sexually transmitted diseases.<br><br>**Outcome:**<br>Prevention of HIV incidence<br><br>**Any significant effect(s):**<br>No. HIV incidence was much the same between groups overall (HR for 0.5% PRO2000 vs placebo: 1.05 [0.82; 1.34], p=0.71).		• Education<br>• Employment<br>• Partner's circumcision status<br>• Condom not used in last sex
**NCT00213083 [5]**<br>**Intervention:**<br>Microbicide gel (Carraguard) plus condoms vs placebo gel plus condoms.<br><br>**Outcome:**<br>Prevention of HIV incidence<br><br>**Any significant effect(s):**<br>No. HIV was not significantly different between groups (adjusted HR: 0.87 [0.69; 1.09]) overall.		
**VOICE [6]**<br>**Interventions:**<br>5 arms: (i) oral TDF (300 mg) and TDF-FTC placebo, (ii) oral TDF-FTC (300 mg of TDF and 200 mg of FTC) and TDF placebo, (iii) oral TDF placebo and oral TDF-FTC placebo, (iv) vaginal 1% TFV gel, or (v) vaginal placebo gel. Standard HIV risk-reduction counselling, individualized adherence counselling, condoms, and hepatitis B immunization were provided.<br><br>Regarding contraception use during the study, the protocol laid out that "All participants will complete monthly follow-up visits for a period of 12 – 33 months and will receive ongoing HIV risk reduction counseling, condoms, and diagnosis and treatment of STIs throughout the course of study participation".<br><br>**Outcome:**<br>Prevention of HIV incidence<br><br>**Any significant effect(s):**<br>No. The interventions did not change HIV incidence significantly. The effectiveness was −49.0% with TDF (HR: 1.49 [0.97, 2.29]), −4.4% with TDF-FTC (HR: 1.04 [0.73, 1.49]), and 14.5% with TFV gel (HR: 0.85 [0.61, 1.21]).		
**HPTN035 [7]**:<br>**Interventions:**<br>4 arms: 0.5% PRO2000 Gel, BufferGel, Placebo Gel, no gel<br><br>Regarding the risk reduction strategies, the study protocol stated that "enrolled participants then complete monthly follow-up visits for the duration of their participation. At each of these visits, participants complete an interval medical and menstrual history and undergo pregnancy testing. HIV/STD risk reduction counseling messages are		

	reinforced if needed and study supplies (i.e., condoms and the assigned study product, if applicable) are provided".<br><br>**Outcome:**<br>Prevention of HIV incidence<br><br>**Any significant effect(s):**<br>No statistically different effects overall: HIV incidence in the 0.5% PRO2000 gel arm versus the placebo gel arm (HR: 0.7, P = 0.10), and versus the no gel arm (HR: 0.67, P = 0.06); the BufferGel versus placebo gel (HR: 1.10, P = 0.63), and no gel (HR: 1.05, P = 0.78); the placebo gel vs no gel arms (HR: 0.97, P = 0.89).						
**Balkus (2016) [8]**	**VOICE [6]**<br>**Interventions:**<br>5 arms: (i) oral TDF (300 mg) and TDF-FTC placebo, (ii) oral TDF-FTC (300 mg of TDF and 200 mg of FTC) and TDF placebo, (iii) oral TDF placebo and oral TDF-FTC placebo, (iv) vaginal 1% TFV gel, or (v) vaginal placebo gel. Standard HIV risk-reduction counselling, individualized adherence counselling, condoms, and hepatitis B immunization were provided.<br><br>**Outcome:**<br>Prevention of HIV incidence<br><br>**Any significant effect(s):**<br>No. The interventions did not change HIV incidence significantly. The effectiveness was −49.0% with TDF (HR: 1.49 [0.97, 2.29]), −4.4% with TDF-FTC (HR: 1.04 [0.73, 1.49]), and 14.5% with TFV gel (HR: 0.85 [0.61, 1.21]).	RCT; (i) sexually active women, (ii) willing to use contraception / not planning to get pregnant	Median: 24<br>IQR: 21-29	**Outcome determination**: Monthly follow-up censored at 1-year<br><br>**Prediction horizon:**<br>1 year	• Age (<25 vs. 25+ yrs)<br>• Married or living with husband or primary partner<br>• Participant earns her own incomes<br>• Number of live births<br>• Alcohol use in the past 3 mo<br>• Partner provides financial or material support<br>• Primary sex partner has other partners<br>• Primary partner is circumcised<br>• Any curable STI<br>• Curable STIs as separate factors<br>• HSV-2 seropositive<br>• participant education level,<br>• primary male partner circumcision status<br>• vaginal sex in the past 4 weeks,<br>• unprotected sex in the past week<br>• number of sex partners in the past 3 months<br>• anal sex in the past 3 months<br>• intravaginal washing with water in the past<br>• intravaginal washing with soap in the past 3 months.	**Only predictors with univariate association with P<0.05 were included.**<br><br>**Variables included:**<br>• Age (<25 vs. 25+ yrs)<br>• Married or living with husband or primary partner<br>• Alcohol use in the past 3 mo<br>• Partner provides financial or material support<br>• Primary sex partner has other partners<br>• Any curable STIs<br>• HSV-2 seropositive<br>• Number of live births	**Cox regression**;<br>Stepwise backward elimination procedures with final model selected based on Akaike information criterion (AIC)<br><br>**Final model:**<br>• Age (<25 vs. 25+ yrs)<br>• Married or living with husband or primary partner<br>• Alcohol use in the past 3 mo<br>• Partner provides financial or material support<br>• Primary sex partner has other partners<br>• Any curable STIs<br>• HSV-2 seropositive
**Peebles (2020) [9]**	**ECHO [10]**<br>**Intervention(s):**<br>3 arms: (i) intramuscular depot medroxyprogesterone acetate (DMPA-IM), (ii) a copper intrauterine device (IUD), (iii) a levonorgestrel (LNG) implant<br><br>**Outcome:**<br>HIV incidence<br><br>**Any significant effect(s):**<br>No. HRs for were 1.04 [0.82, 1.33] (p=0.72) for DMPA-IM compared with copper IUD, 1.23 [0.95, 1.59] (p=0.097) for DMPA-IM compared with LNG implant, and 1.18 [0.91, 1.53] (p=0·19) for copper IUD compared with LNG implant<br><br>On HIV risk reduction, site teams consistently counselled participants that none of the three contraceptive methods being used in the study provided protection against HIV or other STIs and advised women to always use condoms in addition to their contraceptive method.	RCT; (i) sexually active women, (ii) seeking effective contraception	Stratified analysis for 25-35	**Outcome determination**: Follow-up censored at 1-year<br><br>**Prediction horizon:**<br>1 year	• Age (<27 vs. 27+)<br>• Marital/cohabitation status<br>• Weekly alcohol consumption<br>• HIV-1 prevalence (10-15%, 16-20%, 21-25%, 26-30%)<br>• Province (Western Cape, Eastern Cape, KwaZulu-Natal Gauteng, North West)<br>• No. of sex partners in previous 3 months (0 or 1 vs 2+)<br>• Partner has sex with others (No, Yes or do not know)<br>• Condom use (Never or rarely Sometimes, often, or always)<br>• *N. gonorrhoeae*<br>• *C. Trachomatis*<br>• HSV-2 positive<br>• number of previous pregnancies (continuous and categorical),<br>• number of living children (continuous and categorical)<br>• desire for future children<br>• vaginal sex in the past week (Yes/No)<br>• vaginal sex in the past 2 weeks (Yes/No)<br>• number of vaginal sex acts in the past week<br>• vaginal sex during menses in the previous 3 months,<br>• anal sex in the previous 3 months,<br>• partner circumcision status,<br>• partner HIV-1 status,<br>• educational attainment<br>• presence of vaginal discharge<br>• earns own income* (assumed to be considered as one of the candidate predictors since Peebles validated the	**Only predictors with univariate association with P<0.10 were included.**<br><br>**Variables included:**<br>• Age (<27 vs. 27+)<br>• Marital/cohabitation status<br>• Province (Western Cape, Eastern Cape, KwaZulu-Natal Gauteng, North West)<br>• N. gonorrhoeae<br>• C. Trachomatis<br>• HSV-2 positive	**Cox regression**;<br>Stepwise backward elimination procedures with final model selected based on AIC<br><br>**Final model:**<br>• Age (<27 vs. 27+)<br>• Marital/cohabitation status<br>• Province (Western Cape, Eastern Cape, KwaZulu-Natal Gauteng, North West)<br>• *N. gonorrhoeae**<br>• HSV-2 positive*<br><br>* Laboratory-based variables are not included in the modified model

					VOICE score, and this adds up to 25 predictors claimed by the authors)<br>• Receives material and/or financial support from partner*<br><br>*assumed to be considered as one of the candidate predictors since Peebles validated the VOICE score, and this adds up to 25 predictors claimed by the authors)		
**(II) Adolescent girl and young women (AGYW)**							
**Peebles (2020)**		RCT;<br>(i) female<br>(ii) sexually active<br>(iii) seeking effective contraception	Stratified analysis for 18-24	**Same as above**.	**Please refer to the list of candidate predictors above.**	• Reported condom use frequency<br>• Marital/cohabitation status<br>• Number of sex partners in the previous 3 months,<br>• Whether a primary partner<br>• has other sex partners<br>• Alcohol consumption<br>• HIV-1 prevalence<br>• N. gonorrhoeae<br>• C. Trachomatis<br>• HSV-2 positive	• Reported condom use frequency<br>• Number of sex partners in the previous 3 months,<br>• Whether a primary partner<br>• has other sex partners<br>• Alcohol consumption<br>• HIV-1 prevalence<br>• N. gonorrhoeae<br>• HSV-2 positive
**Burgess (2018) [11]**	**CAPRISA004 [12]**<br>**Intervention(s):**<br>Tenofovir gel versus placebo gel<br><br>**HIV risk reduction:**<br>At enrolment and monthly follow-up visits, participants were provided with comprehensive HIV prevention services (HIV pre- and post-test counselling, HIV risk reduction counselling, condoms, and STI treatment), reproductive health services, and assigned study gel.<br><br>**Outcome:**<br>Prevention of HIV incidence<br><br>**Any effect(s):**<br>Yes. Incidence rate ratio in tenofovir gel versus placebo gel was 0.61 (P = 0.017).	RCT;<br>(i) female<br>(ii) sexually active<br>(iii) agree to use a non-barrier form of contraceptive	Stratified analysis for 18-24	**Outcome determination**: Anytime within the follow-up period.<br><br>**Time period of prediction**: the entire follow-up period	**Predictors in the original VOICE score:**<br>• Age (<25 vs. 25+ yrs)<br>• Married or living with husband or primary partner<br>• Alcohol use in the past 3 mo<br>• Partner provides financial or material support<br>• Primary sex partner has other partners<br>• Any curable STIs<br>• HSV-2 seropositive<br><br>And other unspecified predictors of interest, including:<br>• Casual partners in last year	No information.	**Cox regression**;<br>AIC was provided for each model<br><br>**Final model:**<br>• Partner(s) has other partners<br>• HSV-2 positive<br>• Casual partners in last year
**Rosenberg (2020) [13]**	**Rosenberg [14]**<br>**Intervention(s):**<br>Four service delivery models: (i) standard of care, (ii) Youth-Friendly Health Services (YFHS), (iii) YFHS+behavioural intervention and (iv) YFHS+BI+conditional cash transfer<br><br>**Outcome(s):**<br>HIV and SRH health service utilization<br><br>**Any effect(s):**<br>No effects on HIV incidence were reported. However, 26%, 78%, 80%, and 89% of participants received male or female condoms at least once in models (i)-(iv), respectively. Based on clinical data, 72%, 96%, 100%, and 96% of participants received an HIV test at least once in models (i)-(iv).	Quasi-experimental;<br><br>(i) female<br>(ii) 15 to 24 yrs old,<br>(iii) in the clinic's catchment area,<br>(iv) sexually active	15-19 yrs old (58.7%)<br><br>20-24 yrs old (41.3%)	**Outcome determination**: Follow-up at 1-year.<br><br>**Prediction horizon:**<br>1 year	**Predictors in the original VOICE score:**<br>• Age (<25 vs. 25+ yrs)<br>• Married or living with husband or primary partner<br>• Alcohol use in the past 3 months<br>• Partner provides financial or material support<br>• Primary sex partner has other partners<br>• Self-reported vaginal discharge (proxy for curable STIs)<br>• Self-reported genital ulcers (proxy for HSV-2 seropositivity)<br><br>**Other candidate predictors**<br>• Being separated, divorced, or widowed<br>• No running water at home<br>• ⩽2 household assets<br>• Being a double orphan<br>• Multiple sexual partners in the last year<br>• Heavy alcohol use<br>• Self-reported vaginal discharge or genital ulcers<br>• Past pregnancy<br>• Partner slept away ⩾3 nights in the last year<br>• Transactional sex<br>• Uncircumcised partner<br>• Perceived partner concurrency<br>• Partner older for > 5 yrs	The authors first performed automatic stepwise backward elimination to determine which VOICE variables to retain using a likelihood ratio test P-value ⩽0.15. They then added in additional candidate predictors with a univariate association of P⩽0.15.<br><br>**Variables included:**<br>• Age<br>• Self-reported genital ulcers<br>• Self-reported vaginal discharge<br>• Primary sex partner has other partners<br>• >5-year partner age difference<br>• Pregnancy history	**Poisson regression;**<br>Model selected using likelihood ratio tests (LRT)<br><br>**Final model:**<br>• Self-reported genital ulcers<br>• Self-reported vaginal discharge<br>• >5-year partner age difference<br>• Pregnancy history

					• Partner known to be HIV positive	• Divorced, separated, or widowed<br>• ⩾2 sexual partners in the last year<br>• Transactional sex	
**(III) General population**							
**Kagaayi (2014) [15]**	No intervention; Rakai Community Cohort Study (RCCS) [16]	Cohort;<br><br>(i) 15–49 years old;<br>(ii) sexually active<br>(iii) living in Rakai district	**Mean:**<br>27.0 (F)<br>28.3 (M)<br><br>**SD:**<br>7.8 (F)<br>8.0 (M)	**Outcome determination**: Annual follow-up<br><br>**Prediction horizon:**<br>1 year	• Age<br>• Marital status<br>• Education<br>• Number of sexual partners in the last year<br>• Frequency of condom use<br>• Use of alcohol before sex by either partner<br>• Casual sex<br>• Transactional sex<br>• Concurrent sexual partners<br>• Self-perception of exposure to HIV or perception of exposure by partner<br>• Genital ulcer symptoms<br>• Men's circumcision status<br>• Use of hormonal contraception by women<br>• HIV testing and counselling in the previous 12 months<br>• Community type (trading centre versus village),<br>• Whether one migrated to the community within the previous 2 years<br>• Community HIV prevalence<br>• High risk occupation<br>• Partner in a high-risk occupation<br>• Unknown partner's HIV status	All predictors were included in the initial model	**Cox regression;**<br>Stepwise backward elimination procedures selecting model that minimises the AIC minimisation<br><br>**Final model:**<br><br>**Men**<br>• Age<br>• Education<br>• Circumcision status<br>• Number of sexual partners,<br>• Alcohol consumption by self or partner<br>• Genital ulcers<br>• Being unaware of a partner's HIV status<br>• Community type<br>• Having a partner with a high-risk employment<br>• Community type<br>• Community HIV prevalence<br><br>**Women**<br>• Age,<br>• Marital status,<br>• Education,<br>• Number of sex partners,<br>• Having a new sex partner,<br>• Alcohol consumption by self or partner<br>before sex,<br>• Having concurrent relationships,<br>• Being employed in a high-risk occupation,<br>• Having genital ulcers,<br>• Community HIV prevalence,<br>• Perceiving oneself or partner to have been exposed to HIV infection
**Balzer (2020) [17]**	**Sustainable East Africa Research in Community Health (SEARCH) [18]**:<br>**Intervention(s):**<br>Universal antiretroviral therapy (ART) with annual population testing and a multi-disease, patient-centred strategy. Only the intervention arm was included in this analysis.<br><br>**Outcome(s):**<br>Prevention of HIV incidence and improvement of community health<br><br>**Any effect(s):** No significant effect on HIV incidence (relative risk: 0.95 [0.77, 1.17])	RCT;<br>(i) 15+ years old<br>(ii) community residents	15–24 (39%)<br><br>25–34 (20%)<br><br>35–44 (15%)<br><br>45–54 (11%)<br><br>55+ (16%)	**Outcome determination**: Annual follow-up<br><br>**Prediction horizon:**<br>1 year	**DEMOGRAPHY**<br>• Age<br>• Sex<br>• Marital status<br>• Polygamy<br>• Familial relation to head-of-household<br>• Education<br>• Occupation strata<br>• Student<br>• Transportation<br>• Fisherman or fishmonger<br>• Bar worker<br>• Hotel worker<br>• Shopkeeper<br>• Alcohol use<br>• Region	**Method (1) "Risk group approach":**<br>• women aged 15–24 years<br>• individuals with spouses who were living with HIV<br>• alcohol users<br>• widow(er)s<br>• persons employed in transportation<br>• bar worker<br>• fisherman<br><br>**Method (2) "Model-based approach":**<br>All candidate predictors with univariable	**Method (1) "Risk group approach":** the risk score is computed as the sum of the groups (list on the left) to which an individual belongs.<br><br>**Method (2) "Model-based approach":** a logistic model with forward and backward stepwise selection.<br><br>**Method (3) "Machine-learning approach":** Super-learner ensemble method [17]

	**MOBILITY**<br>• Immigrant<br>• Baseline stable resident<br>• Mobile resident<br>• Shifted residence<br>• Nights away<br>**HEALTH**<br>• Health fair attendance<br>• Contraceptive use<br>• Pregnant<br>• Live birth<br>• Male circumcision<br><br>**SPOUSES**<br>• Unknown status<br>• Serodiscordant<br>• Serodiscordant and male (Spouse is HIV-infected and male)<br>• Serodiscordant and circumcision (Spouse is HIV-infected and male partner is not circumcised)<br>• Serodiscordant and polygamous (Spouse is HIV-infected and the marriage is polygamous)<br>• Serodiscordant and unsuppressed (Spouse is HIV-infected with HIV RNA level >500 copies/mL)<br><br>**HOUSEHOLD FACTORS**<br>• Wealth<br>• HIV-unknown adult (At least 1 adult whose HIV status is unknown in the household)<br>• HIV-infected adult (At least 1 HIV-infected adult in the household)<br>• HIV-infected adult of the opposite sex (At least 1 adult of the opposite sex and HIV-infected in the household)<br><br>**INTERACTIONS**<br>• Young woman (Woman aged 15-24 years)<br>• Female bar worker<br>• Wealthy male<br>• Young pregnancy (Woman aged 15-24 years and reporting current pregnancy)<br>• Young mother (Woman aged 15-24 years and reporting at least 1 live birth in the past year)	association of P<0.05 were included<br><br>**Method (3) "Machine-learning approach":** All candidate predictors

**Roberts (2021) [19]**	No intervention. Africa Centre Demographic Information System (ACDIS) cohort [20]	Cohort;<br>(i) Resident members aged 15+ years old	**INDIVIDUAL-LEVEL**<br>• age<br>• sex<br>• marital status<br>• employment<br>• education<br>• socioeconomic status<br>• migration history<br>• ever had sex<br>• prior pregnancy/children (women)<br>• contraception use (women)<br>• circumcision status (men)<br>• number of partners in last 12 months<br>• current number of partners<br>• most recent partner's age<br>• used condom at last sex<br>• most recent partner type (causal vs. regular)<br>• most recent partner residence (same household vs. outside of household)<br>**GEOSPATIAL COVARIATES**<br>• local HIV prevalence (by year)<br>• local population prevalence of detectable viremia (by year)<br>• distance to roads<br>• distance to clinics<br>• distance to schools<br>• urban/rural.	All candidate predictors were included	Cox proportional hazards regression models with lasso penalties and time-varying covariates. Penalties selected via cross-validation.

Table S2. Methods for assessing curable sexually-transmitted infections (STIs) and HSV-2 status.

First author (Year)	Score	Dev/ Val	Sources of data	Variable included in the model	Description	Methods of assessment	Enrolment	Ref
**Wand (2012) [1]**	Self-derived	Dev	MIRA	Genital epithelial disruption	Presence of clinically apparent lesion(s) with epithelial disruption at the baseline	-	-	[21]
				Genital discharge	Presence of genital discharge at the baseline	-	-	-
**Wand (2018) [3]**	Self-derived	Dev	MIRA	Diagnosed with STIs (at least one of chlamydia, gonorrhoea, or syphilis) at the baseline	Laboratory tests at enrolment	• PCR for chlamydia<br>• PCR for gonorrhoea<br>• PCR for Trichomonas	• If positive, participants needed to complete treatment before enrolment	[2]
			MDP301		Laboratory tests at enrolment	• PCR for chlamydia<br>• PCR for gonorrhoea<br>• Laboratory test for trichomonas	• At enrolment, women who had clinically suspicious cervical lesion were referred for assessment and excluded from the study	[4, 22]
			NCT00213083		Laboratory tests at enrolment	• Laboratory test for chlamydia, gonorrhoea, syphilis	-	[5]
			VOICE		Laboratory tests at enrolment	• Urine strand displacement amplification (SDA) for chlamydia<br>• Urine SDA for gonorrhoea<br>• Serology test for syphilis<br>• Rapid test for trichomonas	• At enrolment, women tested positive for STIs were offered treatment and might be enrolled after treatment is completed and symptoms have resolved within 56 days of obtaining informed consent.	[6]
			HPTN 035		Laboratory tests at enrolment	• Urine SDA for chlamydia<br>• Urine SDA for gonorrhoea<br>• Serology test for syphilis<br>Wet mount for trichomonas	• Women tested positive for STIs were not enrolled unless treatment was completed, and all symptoms had resolved within 30 days of obtaining informed consent for screening.	[7]
**Balkus (2016) [8]**	VOICE	Dev	VOICE	STIs at baseline	Laboratory tests at enrolment	• Urine SDA for chlamydia<br>• Urine SDA for gonorrhoea<br>• Serology test for syphilis<br>• Rapid test for trichomonas	• At enrolment, women tested positive for STIs were offered treatment and might be enrolled after treatment is completed and symptoms have resolved within 56 days of obtaining informed consent.	[6]
				HSV-2	Laboratory tests at enrolment	• Serology test for HSV-2		[6]
		Val	HPTN 035	STIs at baseline	Laboratory tests at enrolment	• Urine SDA for chlamydia<br>• Urine SDA for gonorrhoea<br>• Serology test for syphilis<br>• Wet mount for trichomonas	• Women tested positive for STIs were not enrolled unless treatment was completed, and all symptoms had resolved within 30 days of obtaining informed consent for screening.	[23]
				HSV-2	Laboratory tests at enrolment	• Serology test for HSV-2		[23]
		Val	FEM-PrEP	STIs at baseline	Laboratory tests at enrolment	• PCR for chlamydia<br>• PCR for gonorrhoea<br>• Serology test for syphilis<br>• Wet mount for trichomonas		[24]
				HSV-2	Laboratory tests at enrolment	• Serology test for HSV-2		[24]

**Balkus (2018) [25]**	VOICE	Val	ASPIRE	STIs at baseline	Laboratory tests	• PCR for chlamydia<br>• PCR for gonorrhoea<br>• Serology test for syphilis<br>• Rapid test for trichomonas	[26]
				No HSV-2 collected	-	-	-
**Burgess (2017) [27]**	VOICE	Val	FACTS 001	Self-reported STIs	Syndromic management assessed by study staff`	-	
				HSV-2	Laboratory tests	• HSV-2 status established at enrolment according to a testing algorithm in the protocol. Incident cases were confirmed by HSV Western blot.	[28]
**Burgess (2018) [11]**	VOICE + self	Val + Dev	CAPRISA 004	STIs at baseline	Self-report and syndromic management	-	
				HSV-2	Laboratory tests	• Serology test (IgG ELISA) for HSV-2	[29]
**Peebles (2020) [9]**	VOICE + self	Val + Dev	ECHO	STIs at baseline	Laboratory tests	• PCR for chlamydia<br>• PCR for gonorrhoea	[30]
				HSV-2	Laboratory tests	• Serology test for HSV-2	
**Giovenco (2019) [31]**	VOICE	Val	HPTN 068	No curable STIs collected	-	-	
				HSV-2	Laboratory tests	• Serology test (IgG ELISA) for HSV-2	[32]
**Rosenberg (2020) [13]**	VOICE + self	Val + Dev	Girl Power - Malawi	STIs at baseline	Self-reported abnormal vaginal discharge in the last 6 months	-	
				HSV-2	Self-reported genital sores in the last 6 months	-.	
**Ayton (2020) [33]**	VOICE + self	Val	CAPRISA 007	No curable STIs collected	-	-	
				HSV-2	Laboratory tests	• Serology test (IgG ELISA) for HSV-2 measured at baseline, 12 months and 24 months.	[34]
**Kagaayi (2014) [35]**	-	Dev	RCCS	Genital ulcer symptoms	Self-reported genital ulcer symptoms in the past 12 months	-	
**Balzer (2019) [17]**	-	Dev	SEARCH	No STIs collected	-	-	
**Roberts (2021) [19]**	-	Dev	ACDIS	No STIs collected	-	-	

Table S3. Summary table for the risk of bias assessment according to the PROBAST checklist

First author (Year)	Wand (2012)	Wand (2018)	Balkus (2016)	Peebles (2020) 18-24	Peebles (2020) 25-35	Burgess (2018)	Rosenberg (2020)	Kagaayi (2014) Men	Kagaayi (2014) Women	Balzer (2020)	Roberts (2021) Men	Roberts (2021) Women	Wand (2018)	Balkus (2016)	Balkus (2018)	Peebles (2020)	Burgess (2017)	Burgess (2018)	Giovenco (2019)	Ayton (2020)	Rosenberg (2020)
	Article	Article	Article	Article		Poster	Article	Article		Article	Abstract		Article	Article	Letter	Article	Poster	Poster	Article	Article	Art
	Development												Validation								
Participants																					
1.1	Y	Y	Y	Y	Y	Y	Y	Y	Y	Y	Y	Y	Y	Y	Y	Y	Y	Y	Y	Y	Y
1.2	Y	Y	Y	Y	Y	Y	PY	Y	Y	Y	Y	Y	Y	Y	Y	Y	Y	Y	Y	Y	PY
Overall	Low	Low	Low	Low	Low	Low	Low	Low	Low	Low	Low	Low	Low	Low	Low	Low	Low	Low	Low	Low	Low
Predictors																					
2.1	Y	Y	Y	Y	Y	Y	Y	Y	Y	Y	Y	Y	Y	Y	Y	Y	Y	Y	Y	Y	Y
2.2	Y	Y	Y	Y	Y	Y	Y	Y	Y	Y	Y	Y	Y	Y	Y	Y	Y	Y	Y	Y	Y
2.3	Y	Y	Y	Y	Y	Y	Y	Y	Y	Y	Y	Y	N	N	N	Y	PY	Y	N	N	PY
Overall	Low	Low	Low	Low	Low	Low	Low	Low	Low	Low	Low	Low	High	High	High	Low	Low	Low	High	High	Low
Outcome																					
3.1	Y	Y	Y	Y	Y	Y	Y	Y	Y	Y	Y	Y	Y	Y	Y	Y	Y	Y	Y	Y	Y
3.2	Y	Y	Y	Y	Y	Y	Y	Y	Y	Y	Y	Y	Y	Y	Y	Y	Y	Y	Y	Y	Y
3.3	Y	Y	Y	Y	Y	Y	Y	Y	Y	Y	Y	Y	Y	Y	Y	Y	Y	Y	Y	Y	Y
3.4	Y	Y	Y	Y	Y	Y	N	Y	Y	Y	Y	Y	Y	Y	Y	Y	Y	Y	Y	Y	N
3.5	PY	PY	PY	PY	PY	PY	PY	PY	PY	PY	PY	PY	PY	PY	PY	PY	PY	PY	PY	PY	PY
3.6	Y	PN	Y	Y	Y	PY	Y	Y	Y	N	Y	Y	NI	Y	Y	Y	PN	PY	Y	Y	Y
Overall	Low	Low	Low	Low	Low	Low	High	Low	Low	High	Low	Low	Unclear	Low	Low	Low	Low	Low	Low	Low	High
Analysis																					
4.1	N	Y	Y	N	N	NI	N	Y	Y	Y	Y	Y	NI	Y	Y	Y	PY	N	N	N	N
4.2	N	N	Y	Y	N	Y	N	Y	Y	Y	NI	NI	NI	Y	Y	Y	Y	Y	Y	PY	Y
4.3	NI	NI	Y	Y	Y	Y	N	NI	NI	NI	NI	NI	NI	Y	Y	Y	N	Y	N	N	N
4.4	NI	NI	N	Y	Y	Y	N	Y	Y	N	Y	Y	NI	N	Y	Y	N	Y	N	N	N
4.5	N	N	N	N	N	NI	N	Y	Y	N	Y	Y									
4.6	Y	Y	Y	Y	Y	Y	PY	Y	Y	N	Y	Y	NI	Y	Y	Y	Y	Y	Y	Y	Y
4.7	NI	N	NI	Y	Y	NI	NI	Y	Y	NI	Y	Y	NI	Y	Y	Y	Y	Y	Y	PY	Y
4.8	N	Y	N	Y	Y	N	N	N	N	N	Y	Y									
4.9	Y	Y	Y	Y	Y	Y	Y	Y	Y	NI	NI	NI									
Overall	High	High	High	Low	High	Unclear	High	Unclear	Unclear	Unclear	Unclear	Unclear	Unclear	High	Low	Low	High	Low	High	High	High
Overall	High	High	High	Low	High	Unclear	High	Unclear	Unclear	Unclear	Unclear	Unclear	Unclear	High	High	Low	High	Low	High	High	High

To identify information beyond what were given by the risk score articles, we referred to the original sources of data, including the clinical trial protocols, and the methodological articles.

able S4. Summary table on the concerns for applicability according to the PROBAST checklist

Wand (2012)	Wand (2018)	Balkus (2016)	Burgess (2018)	Peebles (2020) 18-24	Peebles (2020) 25-35	Rosenberg (2020)	Kagaayi (2014) Men	Kagaayi (2014) Women	Balzer (2019)	Roberts (2021) Men	Roberts (2021) Women	Wand (2018)	Balkus (2016)	Balkus (2018)	Burgess (2017)	Burgess (2018)	Peebles (2020)	Giovenco (2019)	Ayton (2020)	Rosenberg (2020)
Development												Validation								
(I) Participants																				
Low	Low	Low	Low	Low	Low	Low	Low	Low	Low	Low	Low	Low	Low	Low	Low	Low	Low	Low	Low	Low
(II) Predictors																				
Low	High	High (incl STIs) Low (excl STIs)	High (incl HSVs) Low (excl HSVs)	High (incl STIs) Low (excl STIs)	High (incl STIs) Low (excl STIs)	Low	Low	Low	High	Low	Low	Low	High (incl STIs) Low (excl STIs)	High	High	High	High (incl STIs)	High	High	Low
(III) Outcome																				
Low	High	Low	Low	Low	Low	Low	Low	Low	Low	Low	Low	Uncertain	Low	Low	High	Low	Low	Low	High	Low
Overall																				
Low	High	High (full) Low (excl STIs)	High (incl STIs) Low (excl STIs)	High (incl STIs) Low (excl STIs)	High (incl STIs) Low (excl STIs)	High	Low	Low	Low	Low	Low	Uncertain	High (incl STIs) Low (excl STIs)	High	High	High	High (incl STIs)	High	High	High

Table S5. Summary of missing data and loss-to-follow-up

Author, Year	Ayton 2020	Burgess 2017	Burgess 2018	Balkus 2016			Balkus 2018	Balzer 2020	Kagaayi 2014	Giovenco 2019	Peebles 2020	Roberts 2021	Rosenberg 2020	Wand 2012	Wand 2018
Cohort name	CAPRISA 007	FACTS 001	CAPRISA 004	VOICE	HPTN035	FEM-PrEP	ASPIRE	SEARCH	RCCS	HPTN068	ECHO	ACDIS	Girl Power	MIRA	Multiple
(I) Enrollment															
Total enrolled (excl ineligible if information available)	1049	NI	NI	5007	3101	2120	2629	NI	21957	2533	5670	NI	1000	NI	NI
(II) Analysis															
Imcomplete data	78	944	13	38	NI	NI	NI	NI	NI	355	99	NI	172	NI	NI
Loss to follow up	40	NI	NI	142	NI	NI	90	NI	8677	30	97	NI	NI	NI	NI
Included in the final analysis	971	1115	431	4834	2848	1804	2539	75558	13280	2178	5573	19556	795	1485	8982
% analysed	92.6%	NI	NI	96.5%	91.8%	85.1%	96.6%	NI	60.5%	86.0%	98.3%	NI	79.5%	NI	NI
(III) Handling missing data															
Missing predictors	Imputed	All predictors collected	All predictors collected	N/A (Develop-ment study)	NI	NI	NI	N/A (Develop-ment study)	N/A (Develop-ment study)	NI	All predictors collected	N/A (Develop-ment study)	All predictors collected	N/A (Develop-ment study)	N/A (Develop-ment study)
Missing baseline or outcome data	NI	NI	NI	Missing <5%	NI	NI	NI	Imputed	Imputed	NI	Missing <5%	Imputed	NI	NI	NI

Abbreviation: (NI) No information

Table S6. Summary adjusted and unadjusted hazard ratios (HRs)

		Women; sexually active and seeking effective contraception (RCTs)						AGYW only					
		No. of studies	Pooled estimates	95% CI	Between-studies var	I^2	Ref	No. of studies	Pooled estimates#	95% CI	Between-studies var	I^2	Ref‡
Adjusted effects ¶	Younger age§	5	1.62	[1.17, 2.23]	0.0924	53.1%	[3, 8, 9, 11, 27]	-	-	-	-	-	-
	Not married /cohabiting	6	2.33	[1.73, 3.13]	0.0553	41.3%	[1, 3, 8, 9, 11, 27]	1	1.57#	[0.89;3.09]	-	-	[9]
	No. of sex partners	2	1.62	[1.27; 2.07]	0.0061	0.0%	[1, 3]	2	1.76	[1.19; 2.60]	0.0102	0.0%	[9, 11]
	Partners having other partners	3	1.67	[1.04; 2.71]	0.2322	51.3%	[8, 27]	2	2.35	[0.48; 11.53]	0.9476	65.3%	[9, 11]
	Curable STIs	6	1.45	[1.17; 1.79]	0.0290	0.0%	[1, 3, 8, 9, 11, 27]	2	2.14	[1.40; 3.25]	0.0018	0.0%	11,13
	HSV-2	4	1.67	[1.34; 2.09]	0.0076	0.0%	[8, 9, 11, 27]	3	1.77	[1.24; 2.54]	0.0299	16.1%	[9, 11, 13]
Unadjusted effects§	Younger age	3	1.71	[1.31, 2.22]	0.0180	0.0%	[1, 8, 11]	-	-	-	-	-	-
	Not married /cohabiting	4	1.90	[1.25, 2.87]	0.0979	36.7%	[1, 8, 11, 27]	2	0.76	[0.21, 2.75]	0.3974	43.5%	[13, 31]
	No. of sex partners	2	2.02	[1.44; 2.82]	0.0023	0.0%	[8, 27]	1	2.34	[0.78; 7.00]	-	-	13
	Partners having other partners	2	2.18	[1.51; 3.13]	0.0414	0.0%	[8, 27]	2	1.83	[0.97; 3.48]	0.0048	0.0%	13,31
	Curable STIs	3	1.52	[1.04, 2.21]	0.0663	50.7%	[1, 8, 11]	1	3.36#	[1.17, 9.68]	-	-	[13]
	HSV-2	3	1.47	[1.22; 2.59]	0.0002	0.0%	[8, 11, 27]	2	2.85	[1.31; 6.22]	0.0001	0.0%	13,31

¶ Adjusted HR estimates were only available for predictors retained in the final model after model selection.

# For risk factor estimates that were provided by less than two studies, estimates from the single studies were reported.

§ For all age women studies, all used the <25 cut-off, except for Peebles et al., where they used <27 cut-off for the >25 yrs old.

† Fewer studies provided estimates for unadjusted HR than adjusted HR (listed above). For general women studies, Wand (2018)[3], and Peebles[9], did not provide unadjusted HRs; for AGYW studies, Peebles[9], did not provide unadjusted HRs, while Burgess (2018)[11], provided unadjusted estimates for some factors but not all.

‡ Unlike other studies, Rosenberg[13] used incidence rate ratio estimates rather than hazard ratio.

Table S7. HIV incidence and distribution of high-risk group by each risk score

Score name	Dev/ val	First author (Year)	Sources of data	Highest score available	Score	Observed incidence (per 100 py)	Study population		Incident cases	
							% within each score	Cumulative % of high-risk population	% within each score	Cumulative % of incident cases
**(I) Women only (All ages / 25+years old)**										
VOICE	Dev	Balkus (2016)	VOICE	11	9+	14.7	8.0%	8.0%	18.6%	18.6%
					8	11.9	9.2%	17.2%	17.5%	36.1%
					7	10.4	16.3%	33.4%	27.4%	63.5%
					6	5.5	18.9%	52.3%	17.1%	80.6%
					5	5.4	11.3%	63.6%	10.3%	90.9%
					4	1.9	18.2%	81.8%	5.7%	96.6%
					3	1.7	4.1%	85.9%	1.1%	97.7%
					2	0.8	11.0%	96.9%	1.5%	99.2%
					1	4.1	0.6%	97.4%	0.4%	99.6%
					0	0.9	2.6%	100.0%	0.4%	100.0%
VOICE	Val	Balkus (2016)	HPTN035	10	8+	8.8	6.0%	6.0%	15.3%	15.3%
					7	7.8	7.2%	13.2%	16.3%	31.6%
					6	6.0	10.4%	23.7%	18.4%	50.0%
					5	4.9	5.7%	29.3%	8.2%	58.2%
					4	3.1	21.2%	50.6%	19.4%	77.6%
					3	4.8	4.3%	54.9%	6.1%	83.7%
					2	1.4	29.1%	84.0%	12.2%	95.9%
					1	0.0	1.3%	85.3%	0.0%	95.9%
					0	0.9	14.7%	100.0%	4.1%	100.0%
VOICE	Val	Balkus (2016)	FEM-PrEP	4	4	7.3	8.5%	8.5%	11.9%	11.9%
					3	6.5	28.1%	36.6%	35.6%	47.5%
					2	5.4	32.5%	69.1%	35.6%	83.1%
					1	2.0	25.2%	94.2%	11.9%	94.9%
					0	3.8	5.8%	100.0%	5.1%	100.0%
VOICE	Val	Balkus (2018)	ASPIRE	8	8	10.7	1.5%	1.5%	4.2%	4.2%
					7	10.0	8.0%	9.6%	21.1%	25.3%
					6	5.7	15.9%	25.5%	24.2%	49.5%
					5	3.2	9.7%	35.1%	8.4%	57.9%
					4	4.0	23.7%	58.8%	25.3%	83.2%
					3	4.6	6.0%	64.8%	7.4%	90.5%
					2	1.3	27.8%	92.6%	9.5%	100.0%
					1	0.0	1.1%	93.7%	0.0%	100.0%
					0	0.0	6.3%	100.0%	0.0%	100.0%

Score name		First author (Year)	Sources of data	Highest score available	Score	Observed incidence (per 100 py)	Study population		Incident cases	
							% within each score	Cumulative % of high-risk population	% within each score	Cumulative % of incident cases
VOICE	Val	Burgess* (2017)	FACTS001	11	9+	6.1	10.1%	10.1%	13.6%	13.6%
					8	4.3	11.0%	21.2%	11.1%	24.7%
					7	4.9	20.7%	41.9%	23.5%	48.2%
					6	5.6	12.4%	54.3%	16.1%	64.2%
					5	3.6	23.2%	77.5%	19.8%	84.0%
					4	4.7	8.1%	85.6%	8.6%	92.6%
					3	2.0	8.0%	93.5%	3.7%	96.3%
					2	2.5	4.5%	98.0%	2.5%	98.8%
					1	3.6	1.4%	99.4%	1.2%	100.0%
					0	0.0	0.6%	100.0%	0.0%	100.0%
VOICE	Val	Burgess¶* (2018)	CAPRISA 004	11	11	17.0	0.9%	0.9%	-	-
					10+	>30	7.7%	8.6%	-	-
					9+	13.0	15.3%	23.9%	-	-
					8+	7.5	22.0%	45.9%	-	-
					7+	6.0	29.2%	75.2%	-	-
					6+	5.2	8.6%	83.8%	-	-
					5+	5.2	11.6%	95.4%	-	-
					4+	5.5	2.6%	97.9%	-	-
					3+	8.0	1.9%	99.8%	-	-
					2+	0.0	0.2%	100.0%	-	-
					1+	0.0	0.0%	100.0%	-	-
					0+	0.0	0.0%	100.0%	-	-
Self-derived	Dev	Peebles¶ (2020)	ECHO	7	7	12.0	0.8%	0.8%	2.8%	2.8%
					6	8.5	16.1%	16.9%	39.9%	42.7%
					5	2.9	41.2%	58.1%	35.9%	78.6%
					4	2.3	28.9%	87.0%	18.6%	97.2%
					3	0.5	8.8%	95.8%	1.4%	98.6%
					2	1.1	4.2%	100.0%	1.4%	100.0%
					1	0.0	0.0%	100.0%	0.0%	100.0%

Score name	Dev/ val	First author (Year)	Sources of data	Highest score possible	Score	Observed incidence (per 100 py)	Study population		Incident cases	
							% within each score	Cumulative % of high-risk population	% within each score	Cumulative % of incident cases
**(II) AGYW**										
VOICE	Val	Giovenco (2019)	HPTN 068	10	10	0.0	0.1%	0.1%	6.1%	6.1%
					9	0.0	0.5%	0.6%	0.0%	6.1%
					8	7.1	1.2%	1.7%	9.1%	15.2%
					7	1.9	6.2%	7.9%	45.5%	60.6%
					6	2.2	14.7%	22.7%	24.2%	84.9%
					5	0.9	71.2%	93.9%	9.1%	93.9%
					4	2.3	5.4%	99.3%	6.1%	100.0%
					3	0.0	0.1%	99.4%	0.0%	100.0%
					2	12.9	0.6%	100.0%	0.0%	100.0%
VOICE	Val	Ayton (2020)	CAPRISA 007	6	6	0.0	0.1%	0.1%	0.0%	0.0%
					5	8.0	2.6%	2.7%	14.3%	14.3%
					4	1.4	7.2%	9.9%	7.1%	21.4%
					3	1.0	21.2%	31.1%	14.3%	35.7%
					2	1.4	68.8%	99.9%	64.3%	100.0%
					1	0.0	0.0%	99.9%	0.0%	100.0%
					0	0.0	0.1%	100.0%	0.0%	100.0%
Self-derived	Dev	Burgess¶* (2018)	CAPRISA 004	9	9	16.5	1.4%	1.4%	-	-
					8	>30	11.3%	12.7%	-	-
					7	13.0	19.2%	32.0%	-	-
					6	11.0	24.1%	56.0%	-	-
					5	5.3	33.0%	89.0%	-	-
					4	8.5	5.5%	94.5%	-	-
					3	0.0	5.2%	99.7%	-	-
					2	0.0	0.3%	100.0%	-	-
					1	0.0	0.0%	100.0%	-	-
					0	0.0	0.0%	100.0%	-	-

Score name	Dev/ Val	First author (Year)	Sources of data	Highest score possible	Score	Observed incidence (per 100 py)	Study population		Incident cases	
							% within each score	Cumulative % of high-risk population	% within each score	Cumulative % of incident cases
Self-derived	Dev	Peebles¶ (2020)	ECHO	11	11	0	0.0%	0.0%	0.0%	0.0%
					10	28.6	0.3%	0.3%	0.7%	0.7%
					9	15.5	0.6%	0.9%	1.7%	2.4%
					8	12.4	1.9%	2.8%	4.3%	6.7%
					7	12.5	3.1%	5.9%	7.1%	13.8%
					6	9.2	5.9%	11.8%	9.8%	23.6%
					5	7.2	18.7%	30.5%	25.0%	48.6%
					4	4.9	33.8%	64.3%	31.2%	79.8%
					3	3.6	20.4%	84.7%	14.3%	94.1%
					2	2.5	10.7%	95.4%	4.8%	98.9%
					1	1.3	4.6%	100.0%	1.1%	100.0%
VOICE	Val	Rosenberg¶* (2020)	Girls Power	11	9+	5.3	-	-	-	-
					8	1.5	-	-	-	-
					7	1.3	-	-	-	-
					6	5.5	-	-	-	-
					5	1.0	-	-	-	-
					4	0.5	-	-	-	-
Self-derived	Dev			5	5	22.8	-	-	-	-
					4	6.0	-	-	-	-
					3	4.5	-	-	-	-
					2	1.8	-	-	-	-
					1	1.3	-	-	-	-
**(III) General population**										
Self-derived (men)	Dev	Kagaayi (2017)	RCCS (men)	280	179+	-	20.0%	20.0%	46.7%	46.7%
					175+	-	5.0%	25.0%	8.4%	55.1%
					169+	-	8.3%	33.3%	8.0%	63.1%
					164+	-	6.7%	40.0%	7.1%	70.2%
					158+	-	10.0%	50.0%	8.5%	78.7%
Self-derived (women)	Dev	Kagaayi (2017)	RCCS (women)	500	325+	-	20.0%	20.0%	40.1%	40.1%
					314+	-	5.0%	25.0%	7.9%	48.0%
					298+	-	8.3%	33.3%	7.6%	55.6%
					286+	-	6.7%	40.0%	7.6%	63.2%
					271+	-	10.0%	50.0%	5.5%	68.7%

Self-derived (men & women)	Dev	Balzer (2020)	SEARCH (Risk group model)	-	-	-	20.0%	20.0%	8.0%	8.0%
					-	-	10.0%	30.0%	0.0%	8.0%
					-	-	10.0%	40.0%	0.0%	8.0%
					-	-	5.0%	45.0%	50.0%	58.0%
	Dev		SEARCH (Logistic model)	-	-	-	20.0%	20.0%	40.0%	40.0%
					-	-	10.0%	30.0%	15.0%	55.0%
					-	-	10.0%	40.0%	13.0%	68.0%
					-	-	5.0%	45.0%	0.0%	68.0%
	Dev		SEARCH (Machine-learning)	-	-	-	20.0%	20.0%	52.0%	52.0%
					-	-	10.0%	30.0%	13.0%	65.0%
					-	-	10.0%	40.0%	9.0%	74.0%
					-	-	5.0%	45.0%	4.0%	78.0%
Self-derived (men)	Dev	Roberts ¶ (2021)	ACDIS (women)	-	5th quintile	2.7	20.0%	20.0%	-	-
					4th quintile	1.6	20.0%	40.0%	-	-
					3rd quintile	0.6	20.0%	60.0%	-	-
					2nd quintile	0.5	20.0%	80.0%	-	-
					1st quintile	0.3	20.0%	100.0%	-	-
Self-derived (men)	Dev	Roberts ¶ (2021)	ACDIS (women)	-	5th quintile	6.5	20.0%	20.0%	-	-
					4th quintile	3.8	20.0%	40.0%	-	-
					3rd quintile	2.9	20.0%	60.0%	-	-
					2nd quintile	1.9	20.0%	80.0%	-	-
					1st quintile	0.7	20.0%	100.0%	-	-

¶ Incidence are presented graphically in Burgess (2018) [11], Rosenberg (2020) [13], Peebles (2020) [9], and Roberts (2021) [19].

* Self-reported STIs histories or symptoms were used as proxies for curable STIs in Burgess (2017) [27] and Rosenberg (2020) [13] and for curable STIs in Burgess (2018) [11].

Figure S1. Risk of bias assessment (A) and concerns for applicability (B) for the model development (i) and validation (ii) studies.

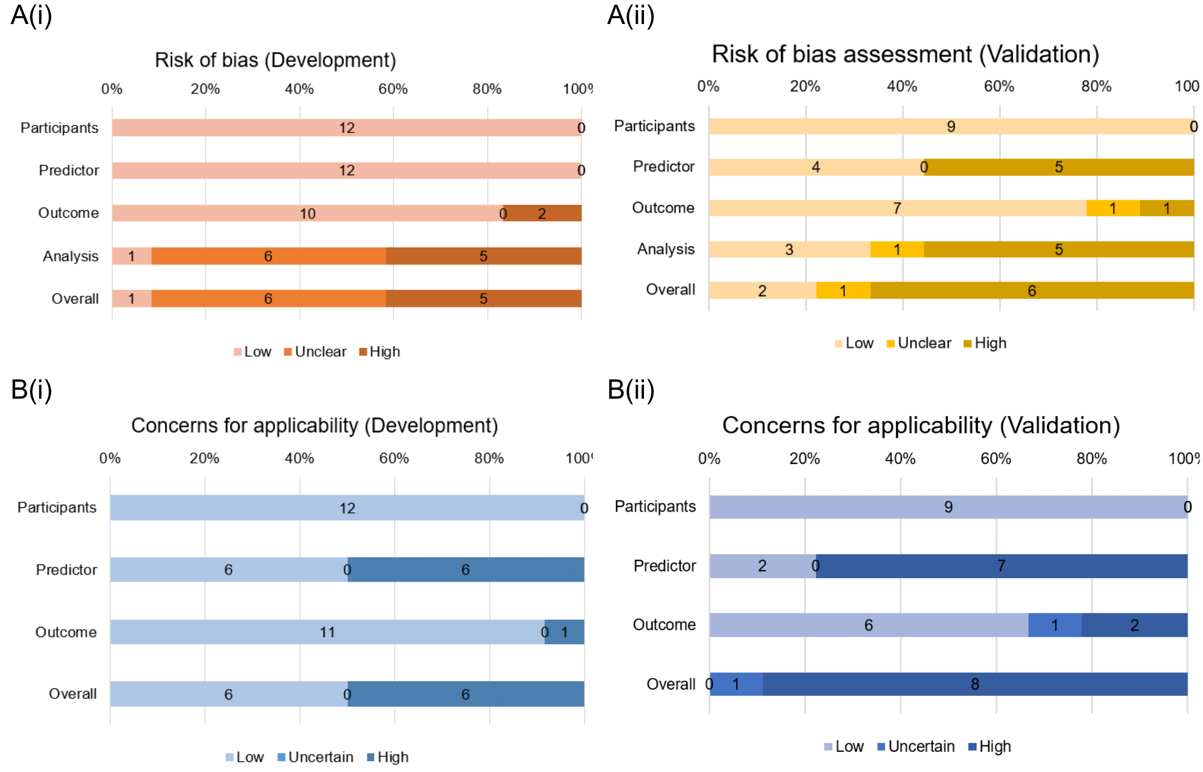

Appendix III. PRIMSA 2020 Abstract Checklist

Topic	No.	Item	Reported?
**TITLE**			
**Title**	1	Identify the report as a systematic review.	Yes
**BACKGROUND**			
**Objectives**	2	Provide an explicit statement of the main objective(s) or question(s) the review addresses.	Yes
**METHODS**			
**Eligibility criteria**	3	Specify the inclusion and exclusion criteria for the review.	Yes
**Information sources**	4	Specify the information sources (e.g. databases, registers) used to identify studies and the date when each was last searched.	No
**Risk of bias**	5	Specify the methods used to assess risk of bias in the included studies.	Yes
**Synthesis of results**	6	Specify the methods used to present and synthesize results.	Yes
**RESULTS**			
**Included studies**	7	Give the total number of included studies and participants and summarise relevant characteristics of studies.	Yes
**Synthesis of results**	8	Present results for main outcomes, preferably indicating the number of included studies and participants for each. If meta-analysis was done, report the summary estimate and confidence/credible interval. If comparing groups, indicate the direction of the effect (i.e. which group is favoured).	Yes
**DISCUSSION**			
**Limitations of evidence**	9	Provide a brief summary of the limitations of the evidence included in the review (e.g. study risk of bias, inconsistency and imprecision).	No
**Interpretation**	10	Provide a general interpretation of the results and important implications.	Yes
**OTHER**			
**Funding**	11	Specify the primary source of funding for the review.	Yes
**Registration**	12	Provide the register name and registration number.	Yes

From: Page MJ, McKenzie JE, Bossuyt PM, Boutron I, Hoffmann TC, Mulrow CD, et al. The PRISMA 2020 statement: an updated guideline for reporting systematic reviews. MetaArXiv. 2020, September 14. DOI: 10.31222/osf.io/v7gm2. For more information, visit: www.prisma-statement.org

Appendix IV. PRISMA 2020 Main Checklist

Topic	No.	Item	Location where item is reported
**TITLE**			
**Title**	1	Identify the report as a systematic review.	p.1
**ABSTRACT**			
**Abstract**	2	See the PRISMA 2020 for Abstracts checklist	
**INTRODUCTION**			
**Rationale**	3	Describe the rationale for the review in the context of existing knowledge.	p.6-7
**Objectives**	4	Provide an explicit statement of the objective(s) or question(s) the review addresses.	p.7
**METHODS**			
**Eligibility criteria**	5	Specify the inclusion and exclusion criteria for the review and how studies were grouped for the syntheses.	p.8
**Information sources**	6	Specify all databases, registers, websites, organisations, reference lists and other sources searched or consulted to identify studies. Specify the date when each source was last searched or consulted.	p.8
**Search strategy**	7	Present the full search strategies for all databases, registers and websites, including any filters and limits used.	p.8; Appendix I
**Selection process**	8	Specify the methods used to decide whether a study met the inclusion criteria of the review, including how many reviewers screened each record and each report retrieved, whether they worked independently, and if applicable, details of automation tools used in the process.	p.8
**Data collection process**	9	Specify the methods used to collect data from reports, including how many reviewers collected data from each report, whether they worked independently, any processes for obtaining or confirming data from study investigators, and if applicable, details of automation tools used in the process.	p.8
**Data items**	10a	List and define all outcomes for which data were sought. Specify whether all results that were compatible with each outcome domain in each study were sought (e.g. for all measures, time points, analyses), and if not, the methods used to decide which results to collect.	p.8; Appendix II
	10b	List and define all other variables for which data were sought (e.g. participant and intervention characteristics, funding sources). Describe any assumptions made about any missing or unclear information.	p.8; Appendix II
**Study risk of bias assessment**	11	Specify the methods used to assess risk of bias in the included studies, including details of the tool(s) used, how many reviewers assessed each study and whether they worked independently, and if applicable, details of automation tools used in the process.	p.8-9
**Effect measures**	12	Specify for each outcome the effect measure(s) (e.g. risk ratio, mean difference) used in the synthesis or presentation of results.	p.9

Topic	No.	Item	Location where item is reported
**Synthesis methods**	13a	Describe the processes used to decide which studies were eligible for each synthesis (e.g. tabulating the study intervention characteristics and comparing against the planned groups for each synthesis (item 5)).	p.9
	13b	Describe any methods required to prepare the data for presentation or synthesis, such as handling of missing summary statistics, or data conversions.	p.9
	13c	Describe any methods used to tabulate or visually display results of individual studies and syntheses.	p.9
	13d	Describe any methods used to synthesize results and provide a rationale for the choice(s). If meta-analysis was performed, describe the model(s), method(s) to identify the presence and extent of statistical heterogeneity, and software package(s) used.	p.9
	13e	Describe any methods used to explore possible causes of heterogeneity among study results (e.g. subgroup analysis, meta-regression).	N/A
	13f	Describe any sensitivity analyses conducted to assess robustness of the synthesized results.	N/A
**Reporting bias assessment**	14	Describe any methods used to assess risk of bias due to missing results in a synthesis (arising from reporting biases).	N/A
**Certainty assessment**	15	Describe any methods used to assess certainty (or confidence) in the body of evidence for an outcome.	N/A
**RESULTS**			
**Study selection**	16a	Describe the results of the search and selection process, from the number of records identified in the search to the number of studies included in the review, ideally using a flow diagram.	p.10
	16b	Cite studies that might appear to meet the inclusion criteria, but which were excluded, and explain why they were excluded.	Figure 1
**Study characteristics**	17	Cite each included study and present its characteristics.	p.10
**Risk of bias in studies**	18	Present assessments of risk of bias for each included study.	Table S4
**Results of individual studies**	19	For all outcomes, present, for each study: (a) summary statistics for each group (where appropriate) and (b) an effect estimate and its precision (e.g. confidence/credible interval), ideally using structured tables or plots.	Figure 2-4; Table 2-3
**Results of syntheses**	20a	For each synthesis, briefly summarise the characteristics and risk of bias among contributing studies.	Figure 2-4
	20b	Present results of all statistical syntheses conducted. If meta-analysis was done, present for each the summary estimate and its precision (e.g. confidence/credible interval) and measures of statistical heterogeneity. If comparing groups, describe the direction of the effect.	Figure 2-4
	20c	Present results of all investigations of possible causes of heterogeneity among study results.	p.12-13
	20d	Present results of all sensitivity analyses conducted to assess the robustness of the synthesized results.	N/A

Topic	No.	Item	Location where item is reported
**Reporting biases**	21	Present assessments of risk of bias due to missing results (arising from reporting biases) for each synthesis assessed.	N/A
**Certainty of evidence**	22	Present assessments of certainty (or confidence) in the body of evidence for each outcome assessed.	N/A
**DISCUSSION**			
**Discussion**	23a	Provide a general interpretation of the results in the context of other evidence.	p.14-16
	23b	Discuss any limitations of the evidence included in the review.	p.15-16
	23c	Discuss any limitations of the review processes used.	p.16
	23d	Discuss implications of the results for practice, policy, and future research.	p.16
**OTHER INFORMATION**			
**Registration and protocol**	24a	Provide registration information for the review, including register name and registration number, or state that the review was not registered.	p.2, 10
	24b	Indicate where the review protocol can be accessed, or state that a protocol was not prepared.	p.2, 10
	24c	Describe and explain any amendments to information provided at registration or in the protocol.	N/A
**Support**	25	Describe sources of financial or non-financial support for the review, and the role of the funders or sponsors in the review.	p. 28
**Competing interests**	26	Declare any competing interests of review authors.	p. 28
**Availability of data, code and other materials**	27	Report which of the following are publicly available and where they can be found: template data collection forms; data extracted from included studies; data used for all analyses; analytic code; any other materials used in the review.	N/A

From: Page MJ, McKenzie JE, Bossuyt PM, Boutron I, Hoffmann TC, Mulrow CD, et al. The PRISMA 2020 statement: an updated guideline for reporting systematic reviews. MetaArXiv. 2020, September 14. DOI: 10.31222/osf.io/v7gm2. For more information, visit: www.prisma-statement.org

References

1. Wand H, Ramjee G. Assessing and evaluating the combined impact of behavioural and biological risk factors for HIV seroconversion in a cohort of South African women. AIDS Care. 2012;24(9):1155-62.

2. Padian NS, van der Straten A, Ramjee G, Chipato T, de Bruyn G, Blanchard K, et al. Diaphragm and lubricant gel for prevention of HIV acquisition in southern African women: a randomised controlled trial. The Lancet. 2007;370(9583):251-61.

3. Wand H, Reddy T, Naidoo S, Moonsamy S, Siva S, Morar NS, et al. A Simple Risk Prediction Algorithm for HIV Transmission: Results from HIV Prevention Trials in KwaZulu Natal, South Africa (2002-2012). AIDS & Behavior. 2018;22(1):325-36.

4. McCormack S, Ramjee G, Kamali A, Rees H, Crook AM, Gafos M, et al. PRO2000 vaginal gel for prevention of HIV-1 infection (Microbicides Development Programme 301): a phase 3, randomised, double-blind, parallel-group trial. The Lancet. 2010;376(9749):1329-37.

5. Skoler-Karpoff S, Ramjee G, Ahmed K, Altini L, Plagianos MG, Friedland B, et al. Efficacy of Carraguard for prevention of HIV infection in women in South Africa: a randomised, double-blind, placebo-controlled trial. The Lancet. 2008;372(9654):1977-87.

6. Marrazzo JM, Ramjee G, Richardson BA, Gomez K, Mgodi N, Nair G, et al. Protocol for: Tenofovir-Based Preexposure Prophylaxis for HIV Infection among African Women. New England Journal of Medicine. 2015;372(6):509-18.

7. Guffey MB, Richardson B, Husnik M, Makanani B, Chilongozi D, Yu E, et al. HPTN 035 phase II/IIb randomised safety and effectiveness study of the vaginal microbicides BufferGel and 0.5% PRO 2000 for the prevention of sexually transmitted infections in women. Sexually Transmitted Infections. 2014;90(5):363-9.

8. Balkus JE, Brown E, Palanee T, Nair G, Gafoor Z, Zhang J, et al. An Empiric HIV Risk Scoring Tool to Predict HIV-1 Acquisition in African Women. Journal of Acquired Immune Deficiency Syndromes. 2016;72(3):333-43.

9. Peebles K, Palanee-Phillips T, Balkus JE, Beesham I, Makkan H, Deese J, et al. Age-Specific Risk Scores Do Not Improve HIV-1 Prediction Among Women in South Africa. Journal of acquired immune deficiency syndromes (1999). 2020;85(2):156-64.

10. Ahmed K, Baeten JM, Beksinska M, Bekker L-G, Bukusi EA, Donnell D, et al. HIV incidence among women using intramuscular depot medroxyprogesterone acetate, a copper intrauterine device, or a levonorgestrel implant for contraception: a randomised, multicentre, open-label trial. The Lancet. 2019;394(10195):303-13.

11. Burgess EK, Yende-Zuma N, Castor D, Karim QA. An age-stratified risk score to predict HIV acquisition in young South African women. Topics in Antiviral Medicine. 2018;26 (Supplement 1):419s.
12. Abdool Karim Q, Abdool Karim SS, Frohlich JA, Grobler AC, Baxter C, Mansoor LE, et al. Effectiveness and Safety of Tenofovir Gel, an Antiretroviral Microbicide, for the Prevention of HIV Infection in Women. Science. 2010;329(5996):1168-74.
13. Rosenberg NE, Kudowa E, Price JT, Pettifor A, Bekker L-G, Hosseinipour MC, et al. Identifying Adolescent Girls and Young Women at High Risk for HIV Acquisition: A Risk Assessment Tool From the Girl Power-Malawi Study. Sexually Transmitted Diseases. 2020;47(11):760-6.
14. Rosenberg NE, Bhushan NL, Vansia D, Phanga T, Maseko B, Nthani T, et al. Comparing Youth-Friendly Health Services to the Standard of Care Through “Girl Power-Malawi”: A Quasi-Experimental Cohort Study. JAIDS Journal of Acquired Immune Deficiency Syndromes. 2018;79(4):458-66.
15. Kagaayi J, Gray RH, Whalen C, Fu P, Neuhauser D, McGrath JW, et al. Indices to measure risk of HIV acquisition in Rakai, Uganda. PLoS ONE. 2014;9(4).
16. Rakai Health Sciences Program (RHSP). RCCS Overview 2021 [11.05.2021]. Available from: https://www.rhsp.org/research/rccs/rccs-overview.
17. Balzer LB, Havlir DV, Kamya MR, Chamie G, Charlebois ED, Clark TD, et al. Machine Learning to Identify Persons at High-Risk of Human Immunodeficiency Virus Acquisition in Rural Kenya and Uganda. Clinical Infectious Diseases. 2020;71(9):2326-33.
18. Havlir DV, Balzer LB, Charlebois ED, Clark TD, Kwarisiima D, Ayieko J, et al. HIV Testing and Treatment with the Use of a Community Health Approach in Rural Africa. New England Journal of Medicine. 2019;381(3):219-29.
19. Roberts A, Cuadros D, Vandormael A, Gareta D, Barnabas R, Herbst K, et al. Predicting Risk of HIV Acquisition in Rural South Africa Using Geographic Data. Conference on Retroviruses and Opportunistic Infections (CROI) International Antiviral Society–USA 2021.
20. Gareta D, Baisley K, Mngomezulu T, Smit T, Khoza T, Nxumalo S, et al. Cohort Profile Update: Africa Centre Demographic Information System (ACDIS) and population-based HIV survey. Int J Epidemiol. 2021;50(1):33-4.
21. Effectiveness of the Diaphragm for HIV Prevention. https://ClinicalTrials.gov/show/NCT00121459.
22. McCormack S. An international multi-centre, randomised, double-blind, placebo controlled trial to evaluate the efficacy and safety of 0.5% and 2% Pro 2000/5 gels for the prevention of vaginally acquired human immunodeficiency virus (HIV) infections. 2017.

23. HPTN 035 study team. Protocol for: HPTN 035 - Phase II/IIb Safety and Effectiveness Study of the Vaginal Microbicides BufferGel and 0.5% PRO 2000/5 Gel (P) for the Prevention of HIV Infection in Women 2007 [Available from: https://www.hptn.org/sites/default/files/2016-05/HPTN035v3_0.pdf.
24. Van Damme L, Corneli A, Ahmed K, Agot K, Lombaard J, Kapiga S, et al. Protocol for: Preexposure Prophylaxis for HIV Infection among African Women. New England Journal of Medicine. 2012;367(5):411-22.
25. Balkus JE, Brown ER, Palanee-Phillips T, Matovu Kiweewa F, Mgodi N, Naidoo L, et al. Performance of a Validated Risk Score to Predict HIV-1 Acquisition Among African Women Participating in a Trial of the Dapivirine Vaginal Ring. Journal of acquired immune deficiency syndromes (1999). 2018;77(1):e8-e10.
26. Baeten JM, Palanee-Phillips T, Brown ER, Schwartz K, Soto-Torres LE, Govender V, et al. Protocol for: Use of a Vaginal Ring Containing Dapivirine for HIV-1 Prevention in Women. New England Journal of Medicine. 2016;375(22):2121-32.
27. Burgess EK, Delany-Moretlwe S, Pisa P, Ahmed K, Sibiya S, Gama C, et al. Validation of a risk score for HIV acquisition in young African women with facts 001. Topics in Antiviral Medicine. 2017;25 (1 Supplement 1):364s-5s.
28. CONRAD, Studies F-oACfT, Development USAfI. Safety and Effectiveness of Tenofovir Gel in the Prevention of Human Immunodeficiency Virus (HIV-1) Infection in Women and the Effects of Tenofovir Gel on the Incidence of Herpes Simplex Virus (HSV-2) Infection. https://ClinicalTrials.gov/show/NCT01386294; 2011.
29. Abdool Karim SS, Abdool Karim Q, Kharsany ABM, Baxter C, Grobler AC, Werner L, et al. Tenofovir Gel for the Prevention of Herpes Simplex Virus Type 2 Infection. N Engl J Med. 2015;373(6):530-9.
30. The Evidence for Contraceptive options and HIV Outcomes (ECHO) Trial 2017 [Available from: https://gatesopenresearch.s3.amazonaws.com/supplementary/12775/e0dbfab3-d280-4003-860c-30cad2ef5a0c.pdf.
31. Giovenco D, Pettifor A, MacPhail C, Kahn K, Wagner R, Piwowar-Manning E, et al. Assessing risk for HIV infection among adolescent girls in South Africa: an evaluation of the VOICE risk score (HPTN 068). Journal of the International AIDS Society. 2019;22(7):e25359-.
32. Pettifor A, MacPhail C, Selin A, Gómez-Olivé FX, Rosenberg M, Wagner RG, et al. HPTN 068: A Randomized Control Trial of a Conditional Cash Transfer to Reduce HIV Infection in Young Women in South Africa-Study Design and Baseline Results. AIDS and behavior. 2016;20(9):1863-82.

33. Ayton SG, Pavlicova M, Abdool Karim Q. Identification of adolescent girls and young women for targeted HIV prevention: a new risk scoring tool in KwaZulu Natal, South Africa. Scientific reports. 2020;10(1):13017.

34. Ayton SG, Pavlicova M, Tamir H, Abdool Karim Q. Development of a prognostic tool exploring female adolescent risk for HIV prevention and PrEP in rural South Africa, a generalised epidemic setting. Sexually transmitted infections. 2020;96(1):47-54.

35. Kagaayi J. Indices to predict the risk of HIV in Rakai, Uganda: Application to the scale-up of safe male circumcision for HIV prevention. Dissertation Abstracts International: Section B: The Sciences and Engineering. 2021;82(3-B):No Pagination Specified.
